# Transcriptome assembly and candidate genes involved in nutritional programming in the swordtail fish *Xiphophorus multilineatus*

**DOI:** 10.7717/peerj.3275

**Published:** 2017-05-02

**Authors:** Yuan Lu, Charlotte M. Klimovich, Kalen Z. Robeson, William Boswell, Oscar Ríos-Cardenas, Ronald B. Walter, Molly R. Morris

**Affiliations:** 1 Molecular Bioscience Research Group, Department of Chemistry and Biochemistry, Texas State University, San Marcos, TX, USA; 2 Department of Biological Sciences, Ohio University, Athens, OH, USA; 3 Red de Biología Evolutiva, Instituto de Ecología A.C, Xalapa, Veracruz, Mexico

**Keywords:** RNA-Seq, *Xiphophorus multilineatus*, Transcriptome, Energy homeostasis

## Abstract

**Background:**

Nutritional programming takes place in early development. Variation in the quality and/or quantity of nutrients in early development can influence long-term health and viability. However, little is known about the mechanisms of nutritional programming. The live-bearing fish *Xiphophorus multilineatus* has the potential to be a new model for understanding these mechanisms, given prior evidence of nutritional programming influencing behavior and juvenile growth rate. We tested the hypotheses that nutritional programming would influence behaviors involved in energy homeostasis as well gene expression in *X. multilineatus.*

**Methods:**

We first examined the influence of both juvenile environment (varied in nutrition and density) and adult environment (varied in nutrition) on behaviors involved in energy acquisition and energy expenditure in adult male *X. multilineatus*. We also compared the behavioral responses across the genetically influenced size classes of males. Males stop growing at sexual maturity, and the size classes of can be identified based on phenotypes (adult size and pigment patterns). To study the molecular signatures of nutritional programming, we assembled a *de novo* transcriptome for *X. multilineatus* using RNA from brain, liver, skin, testis and gonad tissues, and used RNA-Seq to profile gene expression in the brains of males reared in low quality (reduced food, increased density) and high quality (increased food, decreased density) juvenile environments.

**Results:**

We found that both the juvenile and adult environments influenced the energy intake behavior, while only the adult environment influenced energy expenditure. In addition, there were significant interactions between the genetically influenced size classes and the environments that influenced energy intake and energy expenditure, with males from one of the four size classes (Y-II) responding in the opposite direction as compared to the other males examined. When we compared the brains of males of the Y-II size class reared in a low quality juvenile environment to males from the same size class reared in high quality juvenile environment, 131 genes were differentially expressed, including metabolism and appetite master regulator *agrp* gene.

**Discussion:**

Our study provides evidence for nutritional programming in *X. multilineatus*, with variation across size classes of males in how juvenile environment and adult diet influences behaviors involved in energy homeostasis. In addition, we provide the first transcriptome of* X. multilineatus*, and identify a group of candidate genes involved in nutritional programming.

## Introduction

Nutritional quality and quantity during critical windows in early development can influence health and viability into adulthood (Lucas, 1994; Symonds et al., 2007). The mechanisms that link nutrition during fetal life and infancy to permanent physiological changes that promote disease are poorly understood. To identify individuals that are most likely to benefit from specific disease management strategies, as well as to develop additional measures to delay or prevent progression of obesity associated diseases, a better understanding of the molecular mechanisms involved in nutritional programming is required.

In the current study, we examine nutritional programming in *Xiphophorus multilineatus*. One of the benefits of examining diseases in new species is the potential for uncovering evolved adaptations to particular environmental (i.e., resources during development) and/or physiological (i.e., obesity) conditions. For example, domestic pigs are selected for fast growth and fat deposition but do not develop hyperglycemia (Gerstein & Waltman, 2006; Whitfield, 2003), unlike wild pigs (Dyson et al., 2006). By exploring these adaptations in nontraditional or “evolutionary mutant” models (Albertson et al., 2009), new disease-causing and disease-modifier genes may be identified (Schartl, 2014). In addition, the more complex the pathways involved in a disorder, the more likely there will be genetic variation influencing those pathways. Given that human metabolic disorders involve interactions between complex pathways and environmental factors, it will be important to study species with some of the same complexities observed in humans, to better understanding the various gene by environment interactions of metabolic disorders.

*Xiphophorus* is a freshwater fish genus comprised of 26 species. These fishes live in drainages in eastern Mexico, Guatemala, Belize and Honduras, with most of the described species living in Mexico. *Xiphophorus* species have long served as useful animal models to fish behavioral study and cancer genetic research (Schartl et al., 2013). Their unique pigmentation patterns and phenotypes, such as extension of the caudal fin (swordtail), as well as their ability to produce viable and fertile interspecies hybrids, have allowed researchers to sort out genetic interactions in successive generations. Swordtail fishes exhibit natural variation in traits associated with obesity and metabolic diseases (Kallman & Kazianis, 2006), including variation in the propensity to develop a unique fat deposition phenotype (nuchal hump; Rauchenberger, Kallman & Morizot, 1990). There is also variation within some species in the age/size at which males reach sexual maturity, when growth ceases. This variation is attributed to functional and non-signal transducing versions of *Mc4r* gene as well as the copy number variation of this gene on the Y-chromosome (Lampert et al., 2010; Schartl et al., 2011). The genetically influenced size classes of males within *X. multilineatus* (Y-s, Y-I, Y-II and Y-L, Kallman, 1989) have also been shown to differ in their growth rate responses to diet, with males from one of the four size classes (Y-II males) growing faster on a high quality diet than males from the other three size classes (Morris et al., 2012).

Nutritional programming has been previously demonstrated in *X. multilineatus* (D’Amore, Rios-Cardenas & Morris, 2015; Murphy, Goedert & Morris, 2014). Swordtail fishes are live-bearing and retain fertilized eggs inside the female body, resulting in birth to a brood of free-swimming fry. *Xiphophorus multilineatus* mothers reared on a high-quality diet produced offspring that were larger, grew faster, and were larger at sexual maturity compared to offspring produced from a poor-quality maternal nutrition background (Murphy, Goedert & Morris, 2014). Maternal nutritional quality also influenced the relationship between boldness (feeding rate in the presence of a predator) and aggression in *X. multilineatus*, such that offspring with mothers reared on high quality diets were less bold (D’Amore, Rios-Cardenas & Morris, 2015). Given that boldness allows a fish to feed even in the presence of a predator, sons from high-quality maternal environments that were larger at birth could potentially afford to be less bold and forgo foraging when conditions are dangerous. Another important behavior involved in energy homeostasis that influences energy intake is feeding rate. Feeding rate can be easily quantified in swordtails, is highly repeatable, a good measure of food intake, and correlated with juvenile growth rates in *X. multilineatus* (MR Morris et al., 2016, unpublished data).

To test our hypotheses that nutritional programming would influence behaviors involved in energy homeostasis, we examined the influence of both juvenile and adult environments on the expression of two behaviors involved in energy homeostasis, energy intake (feeding rate) and energy expenditure (movements) in *X. multilineatus*. To test our hypothesis that nutritional programming would influence gene expression in the brain, we performed gene expression profiling using RNA-Seq on brain samples from *X. multilineatus* individuals having different juvenile nutritional backgrounds. We specifically examined brain tissue due to the established role of neurons in the brain (i.e., arcuate nucleus in the hypothalamus) as sensors of energy stores and initiators of behavioral and metabolic responses to help maintain energy homeostasis (Barsh & Schwartz, 2002). We *de novo* assembled a transcriptome to provide transcript sequence reference that represents this species (see Fig. S1). The transcriptome of *X. multilineatus*, in addition to *X. maculatus, X. hellerii* and *X. couchianus* (Schartl et al., 2013; Shen et al., 2016), facilitates robust and accurate comparison among *Xiphophorus* species.

## Material and Methods

### Fish

The *X. multilineatus* used in the nutritional/behavioral study were *F*_2_ laboratory reared from wild-caught females, collected from the Rio Tambaque, San Luis Potosi, MX. All fish used in nutritional/behavioral study were kept and handled in accordance with protocol approved by Ohio University IACUC (NIH Assurance number A3610-01). Males from all four described size classes (*Y-s, Y-I, Y-II and Y-L*; Kallman, 1989), identified by adult male size and pigment patterns, were included in the nutritional/behavioral study. The *X. multilineatus* sequenced in this study for the transcriptome assembly were supplied by the *Xiphophorus* Genetic Stock Center (For contact information, see: http://www.xiphophorus.txstate.edu/). The *X. multilineatus* stock is maintained by sibling inbreeding for over 20 generations. At dissection, fish are anesthetized in an ice bath and upon loss of gill movement are sacrificed by cranial resection. Organ are dissected directly into TRI-Reagent (Sigma Inc. St. Louis) placed in a dry ice-ethanol bath if the RNA is isolated at the time of dissection, or into RNAlater (Ambion Inc.) and kept at −80° for later use. All fish used in transcriptome assembly were kept and samples taken in accordance with protocol approved by IACUC (IACUC2015107711).

### Nutritional/density treatments

Fish were reared in two different juvenile environments, which varied in food quantity and density) and on two different adult diets, which varied in food quantity, and quality (primarily in the amount of protein; Chong et al., 2004). Low quality juvenile environment fish (LQJE, *N* = 23) were housed in group-tanks from birth (densities > 6 fish per liter). Fish in this group were fed Tetramin once a day; high quality juvenile environment fish (HQJE, *N* = 34) were housed individually in 2.5 L tanks after 14 days of age, and were fed Tetramin and brine shrimp, *ad libitum.* For the adult treatments, adult males from both juvenile treatments were individually housed in 2.5 L tanks and placed on one of two adult diets: fish in the low quality adult environment (LQAE, *N* = 33) were given Ken’s Premium Spirulina flakes once a day (crude protein 45.0% min, crude fat 9.0% min, crude fiber 1.6% max, moisture 9.0% max); Fish in the high quality adult environment (HQAE, *N* = 24) received Spirulina flakes once a day, brine shrimp once a day (Brine Shrimp Direct; protein 55%, fat 14%, ash 8.1%, and moisture 7%), and bloodworms (Hikari; crude protein 6.0% min, crude fat 0.5% min, crude fiber 0.9% max, moisture 89.0% max, phosphorous 0.01% min) once a day. The adult treatments were maintained for five weeks. Rooms where fish were housed were maintained at approximately 22 °C on a 13-h: 11-h light/dark cycle.

### Behavioral assays

We examined two behaviors that play key roles in energy homeostasis, feeding rate (energy intake) and movement (energy expenditure) across fish from all of the four combinations of nutritional treatments (HQJE/HQAE; LQJE/HQAE; HQJE/LQAE and LQJE/LQAE). To test for feeding rate, fish were first acclimated to eating the Nishikoi Wheat Germ Koi food pellet for five days during the fourth week of the diet treatment. After this period, fish were fasted for 24 h before being tested for bite rate. The test consisted of placing a floating food pellet in the home tank and counting the number of times the fish took a bite of the pellet over a 5-min period. Bite rate measured in adults is be repeatable, a good indicator of food intake, and correlated with juvenile growth rate (MR Morris et al., 2016, unpublished data). Energy expenditure was measured after four weeks of the adult diet as well. Fish were placed in a 76-l aquarium that was visually divided into 10 equal sections. After a 5-min acclimation period, the number of times the fish moves into a new section of the tank was counted over a 5-min observation period.

Because the response variable feeding rate did not meet the assumptions of normality and homoscedasticity, we used a Generalized Linear Model (GzLM) with a Poisson distribution and a log link to examine the effects of juvenile environment and adult diet as the independent factors and movements as a covariable. In the case of the response variable movement, we used a GzLM with a normal distribution and an identity link, as the data met the assumptions of normality and homoscedasticity. Here we also included juvenile environment and adult diet as the independent factors, but in this case feeding rate was the covariable. In both cases we found the minimal adequate model for each analyses using a model simplification based on the Akaike’s Information Criterion (AIC). Models were discarded based on the criteria of a difference of more than two Akaike points from the minimal model.

### RNA isolation and RNA sequencing

For transcriptome assembly, brain, liver, skin, testis and gonad were dissected from two individual fish for RNA isolation. To examine differences in gene expression due to juvenile environment, we selected four Y-II size class males to control for differences in growth rate strategies across size classes (Morris et al., 2012). Two were from the high quality juvenile environment (HQJE; see above) and two were from the low quality juvenile environment (LQJE; see above). All four males were from the HQ adult diet treatment. Entire brains were dissected from these four animals. Total RNA from these tissue samples was isolated as previously detailed (Lu et al., 2015; Walter et al., 2015) using TRI-Reagent (Sigma Inc., St. Louis, MO, USA). Tissue samples were homogenized in TRI-Reagent followed by addition of 200 µl/ml chloroform and the samples vigorously shaken and subjected to centrifugation at 12,000 g for 5 min at 4 °C. Total RNA was further purified using RNeasy mini RNA isolation kit (Qiagen, Valencia, CA, USA). Residual DNA was eliminated by performing column DNase digestion at 25 °C for 15 min. Total RNA concentration was determined using a Qubit 2.0 fluorometer (Life Technologies, Grand Island, NY, USA). RNA quality was verified on an Agilent Bioanalyzer (Agilent Technologies, Santa Clara, CA, USA) to confirm that RIN scores were above 8.0 prior to sequencing.

RNA sequencing was performed upon libraries constructed using the Illumina TruSeq library preparation system (Illumina, Inc., San Diego, CA, USA). For transcriptome assembly, a sequencing library was constructed for each individual tissue. For differential gene expression analyses between *X. multilineatus* individuals reared in different juvenile environment, sequencing library was constructed for each brain sample. RNA libraries were sequenced as 100bp pair-end fragments using Illumina Hi-Seq 2000 system (Illumina, Inc., San Diego, CA, USA).

### *De novo* transcriptome assembly

After sequencing, sequencing adaptor sequences were removed from raw sequencing reads. The processed reads were subsequently trimmed and filtered based on quality scores by using a custom filtration algorithm that removes low-scoring sections of each read and preserved the longest remaining fragment (Garcia et al., 2012). A total of 286 million pair-end sequencing reads and 149 million un-paired sequencing reads from all sequenced tissues remained for transcriptome *de novo* assembly. Paired-end sequencing reads were first shuffled into one file using velvet (version 1.2.10) shffleSequences_fastq.pl scripts. *De novo* transcriptome assembly was processed in two steps. Velvet v1.2.10 was used to assemble combined paired-end and un-paired reads (Zerbino & Birney, 2008). We first used k-mer sizes from 55 to 73 and compared contigs assemblies produced from different k-mer sizes to identify the assembly with the longest N50 length. Contig sequences shorter than 500 nt were filtered out from velvet output. K-mer size of 73 produced longest contig N50 length of 875 nt and contigs sequences generated by this k-mer length were subjected for transcript assembly. Next, Oases v0.2.08 was employed to perform the final assembly and report putative transcripts and splice variants, with minimum transcript length set at 500 nt and other parameters set at default values (Schulz et al., 2012). A final assembly having 93,668 transcripts with an N50 of 4,224 nt and a total size of 281.7Mb was produced for *X. multilineatus*.

To eliminate differences between the *X. multilineatus* transcriptome and other *Xiphophorus* species (i.e., *X. maculatus*) transcriptome, and to create a level playing field for accessing polymorphisms between *X. multilineatus* and *X. maculatus*, the transcriptome of *X. multilineatus* was aligned to well characterized *X. maculatus* transcriptome using BLASTN (*e*-value < 1E−10; Shen et al., 2016). Custom Perl scripts were used to identify the best hits and extract aligned regions of the two transcriptomes. There were 18,872 best-hit groups (92% of *X. maculatus* reference sequences) with an N50 of 2,609 nt and a total size of 36.9 Mb. These best-hit homologous sequences of *X. multilineatus* transcriptome are used as reference transcriptome for gene expression profiling in this study.

### Identification of variants between *X. multilineatus* and *X. maculatus*

*X. maculatus* reference transcriptome was downloaded from http://ftp.ensembl.org/pub/release-80/fasta/xiphophorus_maculatus/cdna/Xiphophorus_maculatus.Xipmac4.4.2.cdna.all.fa.gz. Five hundred and sixty-nine manually annotated genes were compared with the Ensembl transcriptome and sequences missed from Ensembl assembly were added to the enhanced version of *X. maculatus* transcriptome (https://viewer.xgsc.txstate.edu/data/transcriptomes/). RNA-Seq short sequencing reads of *X. multilineatus* were aligned to the enhanced version of *X. maculatus* transcriptome using Bowtie2 v2.2.4.0 (Bowtie2 reference: fast gapped-read alignment with Bowtie 2; Langmead & Salzberg, 2012). An alignment file was sorted and pileup file was generated using samtools (Li et al., 2009). SNP and InDels were identified using VarScan (*p* < 0.05; Koboldt et al., 2012).

### Assessment of transcriptome assembly completeness

Benchmarking Universal Single-Copy Orthologs (BUSCO) was utilized to assess the completeness of the assembled transcriptome with default parameter (Simao et al., 2015; http://busco.ezlab.org/). The *X. maculatus* homolog gene annotated *X. multilineatus* transcriptome was compared against the vertebrate BUSCO references (vertebrata_odb9).

### Differential gene expression analysis

The trimmed and filtered sequencing reads of *X. multilineatus* from the nutritional and behavioral study were aligned to the *X. multilineatus* transcriptome using Bowtie2 (Langmead & Salzberg, 2012). Custom Perl scripts were used to count sequencing reads that mapped to reference transcript sequences (Lu et al., 2015). Library size calculation, dispersion estimation and differentially expressed genes were analyzed using R statistical package edgeR (Robinson, McCarthy & Smyth, 2010). The genes that were altered at least 2 fold between LQJE and HQJE individuals with a *p*-value less than 0.01 (Log_2_FC ⩾ 1 or Log_2_FC ⩽ −1, *p* ⩽ 0.01).

### Gene Ontology analysis

*Xiphophorus* Ensembl gene IDs were converted to human homolog gene Ensembl gene IDs and gene symbol using R-Bioconductor package BiomaRt. Gene Ontology (GO) term of each gene was retrieved from human genome database (org.Hs.eg.db). All genes that have a GO term were collected as background genes. Over-represented GO terms were tested using hypergeometric test by R-Bioconductor package GOstat (ontology = Biological Process, *p* value cutoff = 0.001, count ≥ 5; Beissbarth & Speed, 2004). Over-represented GO categories were subsequently submitted to Revigo (http://revigo.irb.hr/) for GO term summarization.

**Table 1:** The minimal adequate model (based on the Akaike’s Information Criterion, AIC) for two behaviors involved in energy homeostasis.

Source	Chi square likelihood ratio	*DF*	*P*
(A) Feeding rate (energy intake)			
Intercept	17.63	1	<0.001
Size class	168.81	3	<0.001
Size class*J. Environ	411.84	3	<0.001
Size class*adult diet	91.96	2	<0.001
Size class*movements	131.8	4	<0.001
(B) Movements (energy expenditure)			
Intercept	63.6	1	<0.001
Adult diet	3.29	1	0.07
Size class* adult diet	14.13	4	0.007

**Notes.**

J. environment Juvenile environment Size class genetically influenced adult male size class

**Figure 1:**
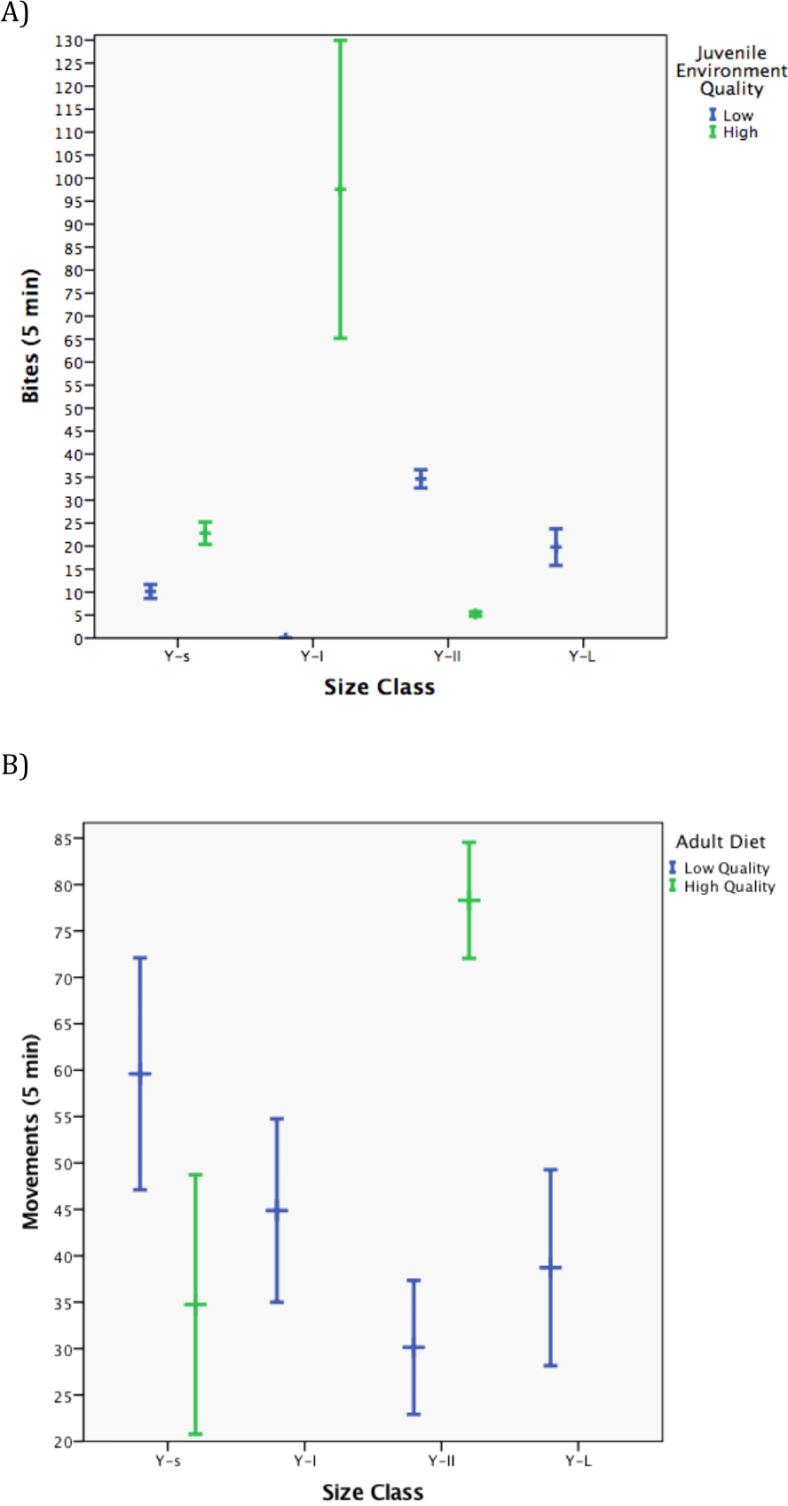
Influence of juvenile and adult environment on behaviors involved in energy homeostasis. (A) Marginal means for energy intake (bites/5 min = feeding rate) across size classes of males (genotypes) reared in two different juvenile environments. (B) Marginal means in energy expenditure (movements = activity level) across size classes of males (genotypes) placed as adults on two different diets.

## Results

### Behaviors

We tested the hypothesis that the quality of juvenile environment and/or adult diet would influence behaviors involved in energy homeostasis using two generalized linear models. Size class (GzLM: likelihood χ^2^_3_ = 168.8, *P* < 0.001), an interaction between size class and adult diet (GzLM: likelihood χ^2^_2_ = 91.96, *P* < 0.001), and an interaction between size class and juvenile environment (GzLM: likelihood χ^2^_3_ = 411.84, *P* < 0.001) influenced energy intake (feeding rate, Table 1A). On examination of the marginal means, Y-II size class males had higher rates of the energy intake behavior (bites/5 min) when reared in LQJE as compared to HQJE, while Y-s and Y-I size class males had higher rates of the energy intake behavior when reared in a HQJE as compared to LQJE (Fig. 1A). An interaction between size class and adult diet influenced energy expenditure (movements, GzLM: likelihood χ^2^_4_ = 14.13, *P* < 0.007, Table 1B). Males from the Y-II size class had a higher rate of energy expenditure in the HQAE as compared to LQAE, and than males from the Y-s size class in the HQAE (Fig. 1B).

**Figure 2:**
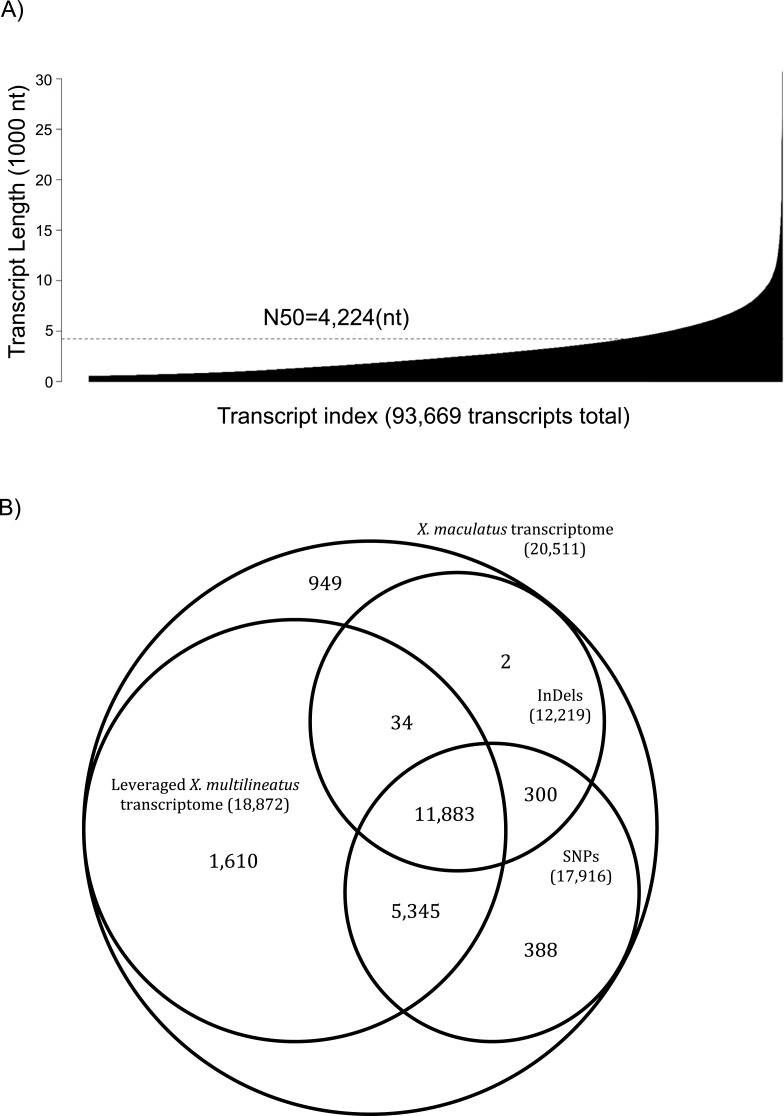
Assembly statistics and sequence variations for the *X. multilineatus* transcriptome. (A) Contigs were first build by Velvet using different k-mer sizes. Subsequently, the assembled contigs were organized to construct transcripts and splicing isoforms using Oases. Oases reported 95,668 putative transcripts, with N50 of 4,224 bp and a size of 281.7 Mb. (B) The *de novo X. multilineatus* transcriptome were aligned to the *X. maculatus* transcriptome using BLASTN. The best reciprocal hits were subsequently determined using custom Perl scripts. Longest best homologous sequences were extracted to form a leveraged *X. multilineatus* transcriptome. 18,872 of 20,511 *X. maculatus* transcripts (or 92% of *X. maculatus* gene models) were presented in *X. multilineatus* transcriptome. The sequencing reads used in *de novo* transcriptome construction was aligned to *X. maculatus* reference transcriptome to distinguish sequence variances between these two species. A total of 409,116 (409,116 SNPs and 41,896 InDels) sequence variants were distinguished from 17,952 genes.

### *X. multilineatus* transcriptome

Next we performed gene expression profiling of brains in adult Y-II males, to capture gene expression differences between Y-II males that are associated with their different juvenile environment. To best represent transcript sequences of *X. multilineatus,* we sequenced mRNA of various *X. multilineatus* tissues, including liver, brain, skin, testis and gonad. The transcriptome for *X. multilineatus* was assembled using a completely *de novo* approach. Velvet and Oases assembling algorithm produced a *X. multilineatus* assembly of 93,668 transcripts that belong to 38,114 locus group with an N50 of 4,224 nt, and a total size of 281.7 Mb (Fig. 2A). One of the major goals of producing transcriptomes beyond this project is to provide *X. multilineatus* reference sequences (https://viewer.xgsc.txstate.edu/data/transcriptomes/). To adopt gene annotation to the assembled transcripts for further gene expression profiling, the *de novo X. multilineatus* transcripts were aligned with the well characterized *X. maculatus* transcriptome. *De novo X. multilineatus* transcriptome was first aligned to *X. maculatus* transcriptome (BLASTN, *e*-value cutoff of 1E−10). The reciprocal best hits were subsequently determined using custom Perl scripts extracting the longest best homologous sequences from *X. multilineatus* assembly. Alignment and trimming steps resulted an *X. multilineatus* transcriptome consisting of 18,872 transcripts and a total size of 36.9 Mbp. Transcriptome assembly completeness assessment using BUSCO showed the *X. multilineatus* is 83.3% completed (81.2% complete and single copy BUSCOs; 2.1% complete and duplicated BUSCOs; 9.2% fragmented BUSCOs, 7.5% missing BUSCOs). This leveraged *X. multilineatus* transcriptome corresponds to 92% of gene number and 68% of total length of Ensembl *X. maculatus* reference transcriptome (Fig. 2B). Additionally, by aligning *X. multilineatus* short-sequencing reads to *X. maculatus* reference sequences, we identified a total of 451,725 variants in which 409,613 were SNPs from 17,916 *X. maculatus* reference transcripts (*p* < 0.05). InDels accounted 41,896 variants that were from 12,219 *X. maculatus* reference transcripts (*p* < 0.05). The overall polymorphisms rate between these two species is about 1 variant in about 81 nt.

### Differential gene expression

**Figure 3:**
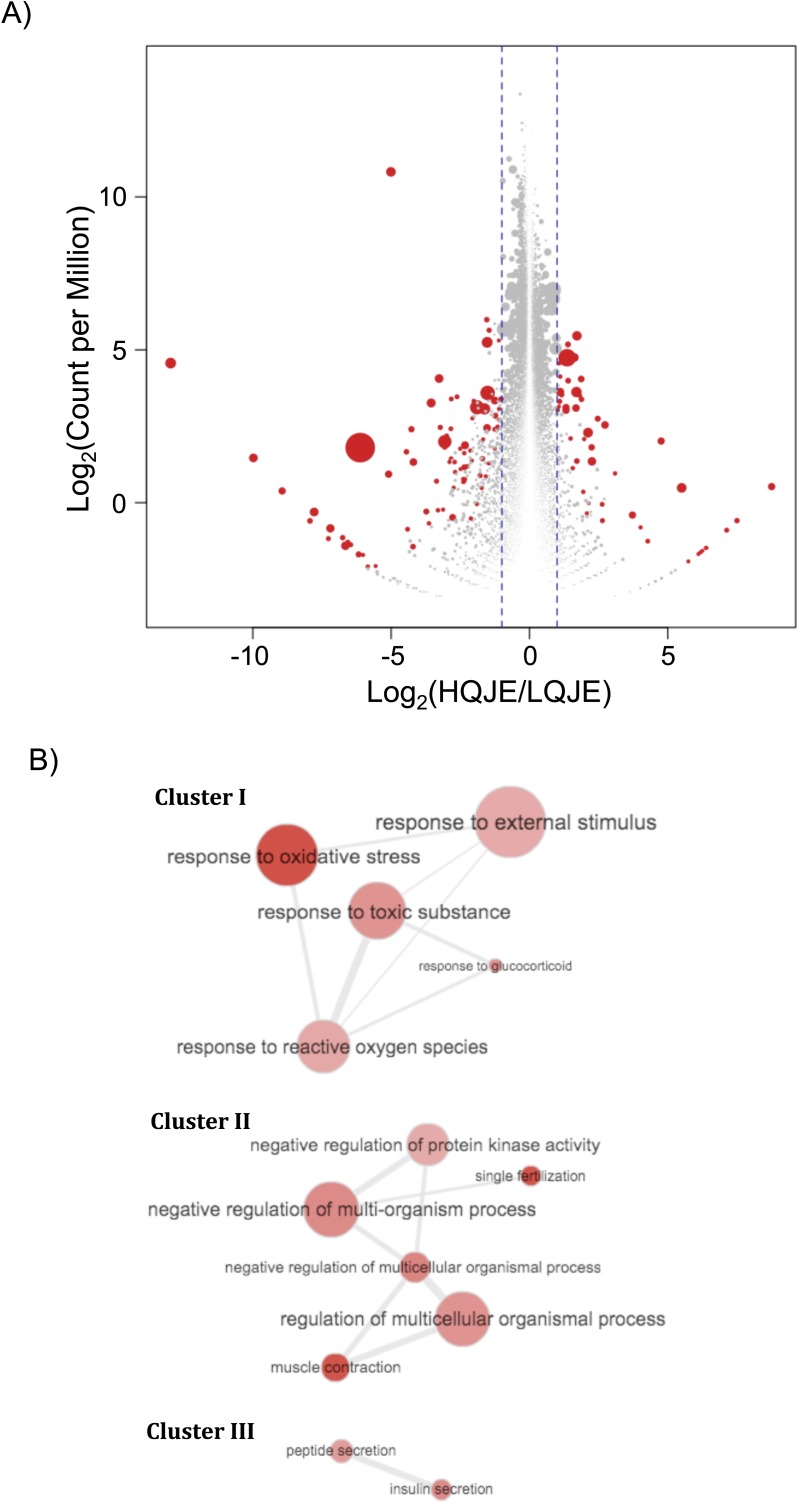
Differential gene expression between HQJE and LQJE Y-II male *X. multilineatus*. (A) Brains of four *X. multilineatus* Y-II males (two were reared in High Quality Juvenile Environment, or HQJE; two reared in Low Quality Juvenile Environment, or LQJE) were sequenced using RNA-Seq for gene expression profiling. Differentially expressed genes were identified using R/Bioconductor package edgeR (Log2(HQJE/LQJE) ≥ 1 or ≤ 1, *p*-value < 0.01). A total of 131 genes were differentially expressed between HQJE males and LQJE males. 83 genes were lower expressed in HQJE fish, and 48 genes were higher expressed in HQJE fish. Gene expression read-counts differences between HQJE Y-II males and LQJE Y-II males (Log2(Count per Million)) were plotted against Log2(HQJE/LQJE). Red color highlights genes that meet statistical cut-off. Size of the dot is a function of p-value (size −1/10 × Log10 *p*-value). (B) Gene Ontology (GO) categories represented by differentially expressed genes were first retrieved. Thirty-one GO categories showed over-representation by using hypergeomatric test (*p* < 0.001; See Table 2). Three functional interrelated GO clusters were identified. These clusters involves functions as response to external stimulus (Cluster I), response to oxidative stress (Cluster I), response to toxic substance (Cluster I), response to reactive oxygen species (Cluster I), and response to glucocorticoid (Cluster I); regulation of multicellular organismal process (Cluster II), negative regulation of multi-organism process (Cluster II), negative regulation of protein kinase activity (Cluster II), negative regulation of multicellular organismal process (Cluster II), muscle contraction (Cluster II) and single fertilization (Cluster II); and peptide secretion (Cluster III), and insulin secretion (Cluster III).

Four Y-II males that differed in their juvenile environment (two HQJE males, two LQJE males), but not adult treatment (all HQAE treatment) were subsequently examined for differences in brain gene expression profiles. A total of 131 genes were differentially expressed between HQJE males and LQJE males (Fig. 3A; Table S1). Eighty-three genes were lower expressed in HQJE fish (Log_2_ (HQJE fish gene expression/LQJE fish gene expression) ≤1, *p* < 0.01), and forty-eight genes were higher expressed in HQJE fish (Log_2_ (HQJE fish gene expression/LQJE fish gene expression) ≥1, *p* < 0.01). Gene Ontology (GO) analysis showed these differentially expressed genes fell into 31 GO categories (count ≥ 5, *p* < 0.001; Table 2). These GO categories formed 3 functionally interrelated clusters, including cluster I (response to external stimulus, response to oxidative stress, response to toxic substance, response to reactive oxygen species, and response to glucocorticoid), cluster II (regulation of multicellular organismal process, negative regulation of multi-organism process, negative regulation of protein kinase activity, negative regulation of multicellular organismal process, muscle contraction and single fertilization) and cluster III (peptide secretion, and insulin secretion; Fig. 3B).

### Discussion

Research addressing the problem of obesity and the associated metabolic disorders in humans has taken a new direction in recent years, one that considers variation across humans in both the genetic propensity for obesity (Bell, Walley & Froguel, 2005; Walley, Asher & Froguel, 2009), as well as early environmental influences such as nutritional programming (Gluckman & Hanson, 2008). The swordtail fish *X. multilineatus* has the potential to be an important model for understanding the molecular mechanisms behind nutritional programming. We detected influences of both the juvenile and adult environments on energy intake behavior, and influences of the adult environment on energy expenditure. In addition, these responses were different across the genetically influenced size classes of *X. multilineatus*. Males from one of the four size classes (Y-II; Kallman, 1989) responded with higher energy intake behavior as adults if reared in a low quality juvenile environment, while males from other size classes responded to the same environment with lower energy intake behavior. Now that we have provided the first transcriptome of *X. multilineatus*, it is possible to identify groups of candidate genes involved in nutritional programming, as well as to examine differences in their expression across the size classes of males that have the opposite behavioral responses to the same environments.

**Table 2:** Over-representation of Gene Ontology categories.

GOBPID	*P* value	OddsRatio	ExpCount	Count	Size	Term	hsapien_gene_name
GO:0032501	8.43E−03	1.69	39.13	51	7,084	Multicellular organismal process	LYST, COL10A1, SMYD1, GIMAP1, AGRP, DUSP2, TOR1A, ZHX2, ACSBG1, FSCN2, ZBTB20, ANPEP, MYLPF, ANXA1, NR4A1, APOE, IRF1, C2orf71, AFF3, STMN1, LPO, ASTL, MPO, MYL1, ATP1A1, NFIB, NME1, NME2, WEE2, ZBTB7B, TNRC6C, KMT2C, SAG, SLC4A2, TK1, TNNC2, TNNI2, TNNT3, TPH1, TRPC6, XDH, CACNA1A, PDGFD, EPX, MYH13, CACNA1H, BSN, SOCS3, ACVR2B, SLC9A3R2, ISG15
GO:0044707	9.75E−03	1.67	33.59	45	6,081	Single-multicellular organism process	LYST, COL10A1, SMYD1, GIMAP1, AGRP, DUSP2, TOR1A, ZHX2, ACSBG1, FSCN2, ZBTB20, ANPEP, MYLPF, ANXA1, NR4A1, APOE, IRF1, AFF3, STMN1, MPO, ATP1A1, NFIB, NME1, NME2, WEE2, ZBTB7B, TNRC6C, KMT2C, SLC4A2, TK1, TNNC2, TNNI2, TNNT3, TPH1, TRPC6, XDH, CACNA1A, PDGFD, EPX, CACNA1H, BSN, SOCS3, ACVR2B, SLC9A3R2, ISG15
GO:0051239	2.73E−03	2.05	14.22	25	2,575	Regulation of multicellular organismal process	ZHX2, ZBTB20, ANPEP, ANXA1, APOE, IRF1, ASTL, ATP1A1, NFIB, NME1, NME2, WEE2, ZBTB7B, TNNC2, TNNI2, TNNT3, TPH1, TRPC6, XDH, CACNA1A, PDGFD, EPX, CACNA1H, ACVR2B, ISG15
GO:0051704	6.43E−03	1.94	13.54	23	2,451	Multi-organism process	MICA, LYST, AGRP, UBR4, ANPEP, APOE, IRF1, STMN1, LPO, MICB, ASTL, MPO, WEE2, KMT2C, SLC4A2, ABCC8, TK1, TRPC6, DNALI1, EPX, CACNA1H, SOCS3, ISG15
GO:0009605	9.40E−03	1.87	13.98	23	2,530	Response to external stimulus	MICA, LYST, XCR1, ANXA1, NR4A1, APOE, IRF1, STMN1, LPO, MICB, MPO, ATP1A1, NFIB, SAG, ABCC8, TK1, TRPC6, CACNA1A, PDGFD, EPX, CACNA1H, SOCS3, ISG15
GO:0006928	9.71E−03	1.97	10.80	19	1,956	Movement of cell or subcellular component	LYST, TOR1A, FAT1, KIF21B, ANXA1, NR4A1, APOE, STMN1, MYL1, ATP1A1, NFIB, TNNC2, TNNI2, TNNT3, TRPC6, DNALI1, PDGFD, EPX, CACNA1H
GO:0045595	7.69E−03	2.14	8.29	16	1,500	Regulation of cell differentiation	SMYD1, ZHX2, ANXA1, APOE, IRF1, NME1, NME2, WEE2, ZBTB7B, TPH1, TRPC6, XDH, CACNA1A, SOCS3, ACVR2B, ISG15
GO:0051241	1.21E−03	2.81	5.56	14	1,007	Negative regulation of multicellular organismal process	ZHX2, ANPEP, ANXA1, APOE, IRF1, ATP1A1, NFIB, NME1, WEE2, TPH1, TRPC6, XDH, EPX, ISG15
GO:0006979	6.15E−05	5.15	2.17	10	393	Response to oxidative stress	TOR1A, ANXA1, APOE, LPO, MICB, MPO, TRPC6, PDGFD, EPX, CHD6
GO:0006936	7.70E−05	5.58	1.80	9	325	Muscle contraction	MYLPF, ANXA1, MYL1, ATP1A1, TNNC2, TNNI2, TNNT3, MYH13, CACNA1H
GO:0003012	3.02E−04	4.61	2.15	9	390	Muscle system process	MYLPF, ANXA1, MYL1, ATP1A1, TNNC2, TNNI2, TNNT3, MYH13, CACNA1H
GO:0010817	1.62E−03	3.59	2.73	9	494	Regulation of hormone levels	ANPEP, ANXA1, ATP1A1, SPCS3, ABCC8, CACNA1A, CACNA1H, SYT7, ACVR2B
GO:0030029	7.60E−03	2.81	3.45	9	625	Actin filament-based process	FAT1, FSCN2, ANXA1, MYL1, ATP1A1, VILL, TNNC2, TNNI2, TNNT3
GO:0048545	8.97E−03	2.93	2.93	8	530	Response to steroid hormone	ACSBG1, NPAS4, ANXA1, NR4A1, ATP1A1, NME1, TK1, SOCS3
GO:0007338	6.97E−05	9.56	0.70	6	126	Single fertilization	ASTL, WEE2, KMT2C, TRPC6, DNALI1, CACNA1H
GO:0009566	2.85E−04	7.29	0.90	6	163	Fertilization	ASTL, WEE2, KMT2C, TRPC6, DNALI1, CACNA1H
GO:0030073	1.65E−03	5.14	1.26	6	228	Insulin secretion	ANXA1, SPCS3, ABCC8, CACNA1A, SYT7, ACVR2B
GO:0009636	2.73E−03	4.63	1.39	6	252	Response to toxic substance	GLYAT, APOE, LPO, MPO, TK1, EPX
GO:0030072	3.76E−03	4.32	1.49	6	269	Peptide hormone secretion	ANXA1, SPCS3, ABCC8, CACNA1A, SYT7, ACVR2B
GO:0002790	4.33E−03	4.19	1.53	6	277	Peptide secretion	ANXA1, SPCS3, ABCC8, CACNA1A, SYT7, ACVR2B
GO:0050678	4.97E−03	4.07	1.57	6	285	Regulation of epithelial cell proliferation	NR4A1, APOE, NFIB, NME1, NME2, XDH
GO:0046879	8.85E−03	3.59	1.78	6	322	Hormone secretion	ANXA1, SPCS3, ABCC8, CACNA1A, SYT7, ACVR2B
GO:0070252	1.47E−04	11.01	0.50	5	91	Actin-mediated cell contraction	MYL1, ATP1A1, TNNC2, TNNI2, TNNT3
GO:0030048	3.57E−04	9.01	0.61	5	110	Actin filament-based movement	MYL1, ATP1A1, TNNC2, TNNI2, TNNT3
GO:0006941	1.37E−03	6.60	0.82	5	148	Striated muscle contraction	MYL1, ATP1A1, TNNC2, TNNI2, TNNT3
GO:0043901	1.63E−03	6.33	0.85	5	154	Negative regulation of multi-organism process	MICB, ASTL, MPO, WEE2, ISG15
GO:0051384	2.15E−03	5.93	0.91	5	164	Response to glucocorticoid	ACSBG1, NPAS4, ANXA1, TK1, SOCS3
GO:0031960	2.85E−03	5.54	0.97	5	175	Response to corticosteroid	ACSBG1, NPAS4, ANXA1, TK1, SOCS3
GO:0000302	7.74E−03	4.33	1.23	5	222	Response to reactive oxygen species	ANXA1, APOE, MPO, TRPC6, PDGFD
GO:0034599	8.63E−03	4.21	1.26	5	228	Cellular response to oxidative stress	ANXA1, MPO, TRPC6, PDGFD, CHD6
GO:0006469	9.76E−03	4.08	1.30	5	235	Negative regulation of protein kinase activity	UBE2C, DUSP2, APOE, WEE2, SOCS3

To initiate the study of candidate genes involved in nutritional programming, we compared gene expression across males from one of the size classes (Y-II) reared in the two different juvenile environments, but on the same high quality adult diet. The differences we detected in gene expression suggest juvenile environment plays a role in altering brain development and function and therefore affected adult behavior and overall energy homeostasis (Table 2). For example, the *agpr* gene encodes Agouti-related protein and is expressed primarily in the hypothalamic arcuate nucleus. *Agrp* acts to increase appetite by binding to *Mc4r* (Chen et al., 2004), and overexpression of *Agrp* in transgenic mice led to obesity (Ollmann et al., 1997). The mRNA concentration of this gene is higher (2^2.33^ fold) in Y-II males reared in the LQ juvenile environment. This result supports the hypothesis that protein restriction during prenatal and postnatal periods can alter the anatomical organization of the melanocortin system (Coupe et al., 2010) in which *Agrp* functions as an important appetite regulator. Additionally, we identified other metabolism related genes including *tk1* (lower expressed in HQJE male), *xdh* (higher expressed in HQJE male), a Phytanoyl-CoA dioxygenase domain containing gene (zgc:174917; higher expressed in HQJE male) and a CNDP dipeptidase 2 ortholog gene (zgc:114181; lower expressed in HQJE male); diabetes related gene *socs3a* (higher expressed in HQJE male); as well as energy and lipid related genes *mogat3a* (higher expressed in HQJE male) and *pnrc2* (lower expressed in HQJE male). Overall, the differential expression of these genes suggests that the brain function, and its relation to metabolism and energy regulation, is affected by quality of juvenile environment.

Additionally, we have identified 18 *Xiphophorus* specific DEGs that showed no homology with human, mouse, rat, chicken, zebrafish, spotted gar, medaka, stickleback, fugu or amazon molly. Annotated genes belong to different functional types, including enzymes (15 genes), G-protein coupled receptor (one gene), growth factor (one gene), ion channel (three genes), kinase (four genes), nuclear receptor (one gene), peptidase (six genes), transmembrane receptor (one gene), phosphatase (two genes), transcription regulator (10 genes), and transporter (eight genes; Table S1).

The behavioral pattern we detected across the Y-II male size class appears to be the most common pattern reported in the literature (Armitage, Taylor & Poston, 2005). Males reared in the low quality juvenile environment from this size class increased their energy intake (measured as feeding rate) as compared to males reared in the high quality juvenile environment. We have detected a relationship between increased feeding rates in adult male fish and increased growth rates as juveniles (MR Morris et al., 2016, unpublished data). Given that males stop growing at sexual maturity, the response of the Y-II adult males to the high quality environment they experienced as juveniles could also lead to increased fat deposition as adults. In humans, this pattern is attributed to genetic influences on eating and metabolic rate (Brophy et al., 2009) as well as behavioral and environmental changes leading to increased energy intake and a lack of physical activity (Jahns, Siega-Riz & Popkin, 2001). However, the influence of supernormal nutrition during early development can also lead to a phenotype that closely resembles the human metabolic syndrome with higher appetites (Armitage et al., 2004; Guo & Jen, 1995), and would appear to fit the pattern we detected across the Y-s and Y-I size classes of swordtail fish. Variation in the *Mc4r* gene plays a key role in energy homeostasis through appetite in several species of fishes (i.e., goldfish Cerdá-Reverter, Schiöth & Peter, 2003; rainbow trout Schjolden et al., 2009; Mexican cavefish Aspiras et al., 2015) as well as in humans (Cole et al., 2010; Adan et al., 2006). Given that the male size classes in *X. multilineatus* are influenced by the different alleles and number of copies of the *Mc4r* gene on the Y chromosome (Lampert et al., 2010), variation across copies of this gene could be involved in producing variation in the responses to nutritional programming. Further examination of gene expression across the various size classes is warranted, and could lead to a better understanding of the mechanisms that are involved in producing variation in response to early nutritional environments. This system, therefore, has the potential to allow us to better understanding the genetic and environmental complexities of susceptibility to obesity and associated metabolic diseases, as well as to help identify best potential genetic targets for treatment across individuals with different developmental histories of nutritional environments.

It will be important to consider the possibility that factors in addition to food availability and quality influenced the differences between individuals reared in the low quality as compared to high quality juvenile environments. The individuals from the low quality juvenile environment (mesocosms) had experience with conspecifics throughout life, and would therefore have experienced many different social interactions as well as competition for limited and lower quality food. In addition, it may be useful at some point to distinguish between nutritional programming and the possibility that the low quality juvenile environments produced allostatic responses, or “wear and tear on the body” of the fish due to the chronic stress of low food quality or high densities. Future studies that rear fish in individually isolated tanks for both juvenile treatments could be conducted and the genes expressed compared to those in the current further examine to test this possibility.

The variation in behavioral responses to nutritional programming we detected could be considered in the context of alternative reproductive tactics, where tradeoffs between different life history traits can select for alternative strategies to maximize fitness. The alternative reproductive tactics (ARTs) that have been identified in *X. multilineatus*, group together the three larger size classes based on their exclusive use of courtship behavior (*Y-I, Y-II and Y-L*), while the smallest size class (*Y-s*) uses both courtship and a coercive sneaking mating behavior (Zimmerer & Kallman, 1989; Morris, Rios-Cardenas & Brewer, 2010). The tradeoff that has been suggested to maintain these ARTs is based on evidence that *Y-s* males reach sexual maturity sooner (Bono, Rios-Cardenas & Morris, 2011), while the courter males that make it to sexual maturity, have a higher mating success (Morris et al., 2012). However, results from the current study add to previous work that the *Y-II* size class may represent an additional ART within the courter males, where the tradeoff maintaining this ART involves the costs and benefits of faster growth. Faster growth can be beneficial for swordtail fishes by increasing the probability of reaching sexual maturity sooner and at a larger size, thus increasing the probability of ever mating. However, costs to growing faster have been identified, and include instable development (Morris et al., 2012), and reduced adult lifespans (Morris et al., 2016). There has been some consideration of life history tradeoffs in relation to variation in human birth weights (Coall & Chisholm, 2003), which can influence the propensity to develop certain metabolic syndromes. We argue that further research into the life history tradeoffs of nutritional programming will add significantly to our understanding of the role this mechanism plays in the development of metabolic syndromes.

## Conclusions

The potential for the perinatal period to program human adult metabolic disorders has been well established (Gluckman, Hanson & Pinal, 2005; Hales & Barker, 2001); however, the mechanisms involved are poorly understood. It will be important to further investigate genetic variation in the responses to nutritional programming, and the results we present here suggest that swordtail fishes will be a model system in which to examine these mechanisms. Our behavioral studies suggest that responses to juvenile environment by a behavior involved in energy intake (feeding rates), as well as responses to adult environment by a behavior that is involved in energy expenditure (movements) will depend on a male’s genetically influenced size class in this species of swordtail fish. This pattern is consistent with our increasing understanding of the involvement of regulatory genetic variation in common human diseases (Maurano et al., 2012). Also, the provision of a transcriptome for this species makes it possible to further examine molecular mechanisms that play a role in nutritional programming. The list of genes that were differentially expressed in the males reared in high versus low quality juvenile environments provides an important starting point for understanding these nutritional programming mechanisms.

##  Supplemental Information

10.7717/peerj.3275/supp-1 Figure S1 Flow chart of transcriptome assembly and differential gene expression analyses A *de novo* transcriptome for *X. multilineatus* was assembled using mRNA isolated from several different tissues. The assembled transcript sequences were aligned to *X. maculatus* reference sequences for annotation. Messenger RNA was isolated from the brain tissues of two Y-II size class males reared in a low quality juvenile environment (LQJE) and two Y-II size class males reared in a high quality juvenile environment (HQJE) and were sequenced. Short sequencing reads were mapped to the *X. multilineatus* transcriptome, and differential gene expression was further analyzed on the quantified read counts between LQJE and HQJE individuals.

10.7717/peerj.3275/supp-2 Table S1 Differentially expressed genes between HQJE and LQJE Y-II males

## Additional Information and Declarations

### Data Availability

The following information was supplied regarding data availability: Raw sequencing data will be publicly available upon manuscript acceptance for publishing. GEO accession number: GSE89561.
