# Supplementary figures and images for: Transcriptome assembly and candidate genes involved in nutritional programming in the swordtail fish Xiphophorus multilineatus
Source: PeerJ. 2017 May 2;5:e3275. doi: 10.7717/peerj.3275 (PMC5417068; doi:10.7717/peerj.3275)

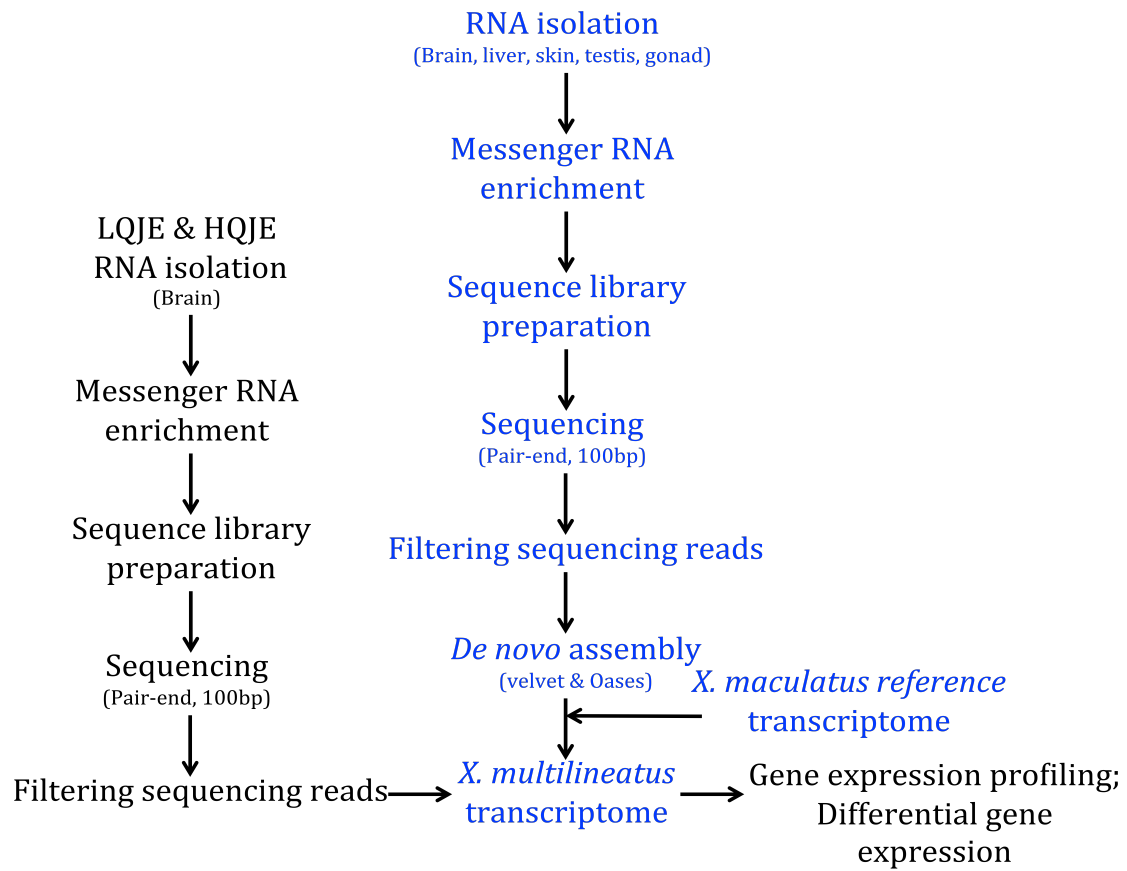

Supplement: Figure S1 — A de novo transcriptome for X. multilineatus was assembled using mRNA isolated from several different tissues. The assembled transcript sequences were aligned to X. maculatus reference sequences for annotation. Messenger RNA was isolated from the brain tissues of two Y-II size class males reared in a low quality juvenile environment (LQJE) and two Y-II size class males reared in a high quality juvenile environment (HQJE) and were sequenced. Short sequencing reads were mapped to the X. multilineatus transcriptome, and differential gene expression was further analyzed on the quantified read counts between LQJE and HQJE individuals. [file peerj-05-3275-s001.pdf]
